# De Novo Mutations in *PDE10A* Cause Childhood-Onset Chorea with Bilateral Striatal Lesions

**DOI:** 10.1016/j.ajhg.2016.02.015

**Published:** 2016-04-07

**Authors:** Niccolò E. Mencacci, Erik-Jan Kamsteeg, Kosuke Nakashima, Lea R’Bibo, David S. Lynch, Bettina Balint, Michèl A.A.P. Willemsen, Matthew E. Adams, Sarah Wiethoff, Kazunori Suzuki, Ceri H. Davies, Joanne Ng, Esther Meyer, Liana Veneziano, Paola Giunti, Deborah Hughes, F. Lucy Raymond, Miryam Carecchio, Giovanna Zorzi, Nardo Nardocci, Chiara Barzaghi, Barbara Garavaglia, Vincenzo Salpietro, John Hardy, Alan M. Pittman, Henry Houlden, Manju A. Kurian, Haruhide Kimura, Lisenka E.L.M. Vissers, Nicholas W. Wood, Kailash P. Bhatia

**Affiliations:** 1 Department of Molecular Neuroscience, UCL Institute of Neurology, WC1N 3BG London, UK; 2 Department of Neurology and Laboratory of Neuroscience, IRCCS Istituto Auxologico Italiano, Department of Pathophysiology and Transplantation, Centro Dino Ferrari, Università degli Studi di Milano, 20149 Milan, Italy; 3 Department of Human Genetics, Donders Centre for Brain, Cognition, and Behavior, Radboud University Medical Center, Geert Grooteplein 10, 6525 GA Nijmegen, the Netherlands; 4 CNS Drug Discovery Unit, Pharmaceutical Research Division, Takeda Pharmaceutical Company Limited, 251-8555 Fujisawa, Japan; 5 Sobell Department of Motor Neuroscience and Movement Disorders, UCL Institute of Neurology, WC1N 3BG London, UK; 6 Department of Neurology, University Hospital Heidelberg, 69120 Heidelberg, Germany; 7 Department of Paediatric Neurology, Donders Centre for Brain, Cognition, and Behavior, Radboud University Medical Center, Geert Grooteplein 10, 6525 GA Nijmegen, the Netherlands; 8 Lysholm Department of Neuroradiology, National Hospital for Neurology and Neurosurgery, WC1N 3BG London, UK; 9 Center for Neurology and Hertie Institute for Clinical Brain Research, Eberhard Karls University, 72076 Tübingen, Germany; 10 Developmental Neurosciences, UCL Institute of Child Health, WC1N 1EH London, UK; 11 Department of Neurology, Great Ormond Street Hospital, WC1N 3JH London, UK; 12 Institute of Translational Pharmacology, National Research Council, 00133 Rome, Italy; 13 Department of Medical Genetics, University of Cambridge, CB2 0XY Cambridge, UK; 14 Neuropediatrics Unit, IRCCS Istituto Neurologico Carlo Besta, 20133 Milan, Italy; 15 Molecular Neurogenetics Unit, IRCCS Istituto Neurologico Carlo Besta, 20133 Milan, Italy; 16 Reta Lila Weston Institute of Neurological Studies, UCL Institute of Neurology, WC1N 3BG London, UK

## Abstract

Chorea is a hyperkinetic movement disorder resulting from dysfunction of striatal medium spiny neurons (MSNs), which form the main output projections from the basal ganglia. Here, we used whole-exome sequencing to unravel the underlying genetic cause in three unrelated individuals with a very similar and unique clinical presentation of childhood-onset chorea and characteristic brain MRI showing symmetrical bilateral striatal lesions. All individuals were identified to carry a de novo heterozygous mutation in *PDE10A* (c.898T>C [p.Phe300Leu] in two individuals and c.1000T>C [p.Phe334Leu] in one individual), encoding a phosphodiesterase highly and selectively present in MSNs. PDE10A contributes to the regulation of the intracellular levels of cyclic adenosine monophosphate (cAMP) and cyclic guanosine monophosphate (cGMP). Both substitutions affect highly conserved amino acids located in the regulatory GAF-B domain, which, by binding to cAMP, stimulates the activity of the PDE10A catalytic domain. In silico modeling showed that the altered residues are located deep in the binding pocket, where they are likely to alter cAMP binding properties. In vitro functional studies showed that neither substitution affects the basal PDE10A activity, but they severely disrupt the stimulatory effect mediated by cAMP binding to the GAF-B domain. The identification of *PDE10A* mutations as a cause of chorea further motivates the study of cAMP signaling in MSNs and highlights the crucial role of striatal cAMP signaling in the regulation of basal ganglia circuitry. Pharmacological modulation of this pathway could offer promising etiologically targeted treatments for chorea and other hyperkinetic movement disorders.

## Main Text

Movement disorders comprise a large clinically and genetically heterogeneous group of disorders, which can be subdivided into various clinical entities, including dystonia and chorea. Although monogenic causes are overall rare, mutations in greater than >200 genes are known to cause either an isolated movement disorder or a syndromic form of movement disorders.^1^, ^2^, ^3^ However, in total, mutations in these genes only explain a small proportion of cases, suggesting that mutations in more genes await discovery.

Chorea is a hyperkinetic movement disorder clinically characterized by continuous and brief involuntary movements, which flow from one body part to another and are unpredictable in terms of timing, speed, and direction. Chorea is a major feature of several inherited neurological disorders.^4^ Functional dysregulation of striatal GABAergic medium spiny neurons (MSNs), which form the main output projections from the basal ganglia, is considered to underlie the pathophysiology of the choreic movements.^5^

**Figure 1:**
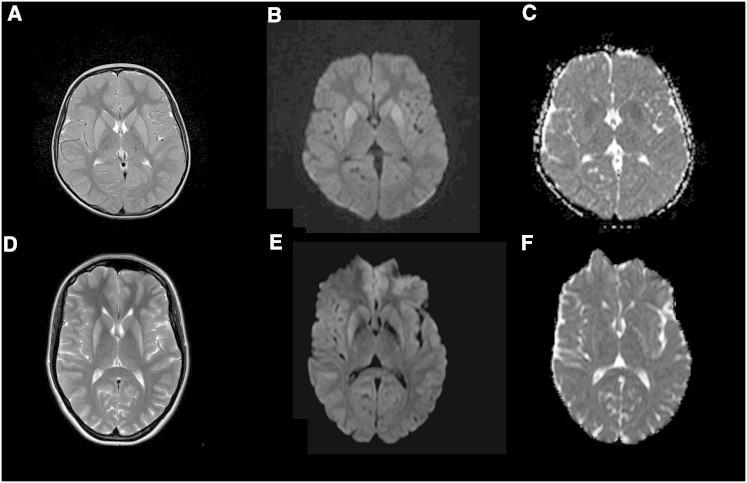
MRI Features Associated with Dominant *PDE10A* Mutations Axial MRI of individuals 1 (A–C) and 2 (D–F). T2-weighted imaging (A and D) and diffusion-weighted imaging (DWI; B and E) showed increased signal intensity within the striatum. In individual 1, the putamen and caudate nucleus appeared slightly swollen (A), and high signal on DWI (B) was confirmed to represent abnormal restricted diffusion on the ADC map (C). In individual 2, the abnormal signal was principally located in the postero-lateral putamina, which also appeared atrophic (D). There was no corresponding restriction of diffusion on the ADC map (F), and appearances suggested a more chronic disease stage.

We have identified three European-descent individuals affected by a similar childhood-onset movement disorder predominantly characterized by chorea and bilateral striatal abnormalities on cerebral MRI. The main clinical and radiological features of the three individuals are presented in Table 1. In brief, all three individuals presented in childhood (age of onset between 5 and 10 years) with a scarcely progressive movement disorder dominated by chorea. Developmental milestones were normal, and there were no other major neurological features, in particular intellectual disability or cognitive decline. Given these clinical features and the absence of a significant progression of symptoms, a diagnosis of benign hereditary chorea (BHC [MIM: 118700])^6^ was initially considered. However, within the striatum in all three individuals, brain MRI consistently showed bilateral T2 hyperintensity (Figure 1), which is an atypical finding for BHC.

**Figure 2:**
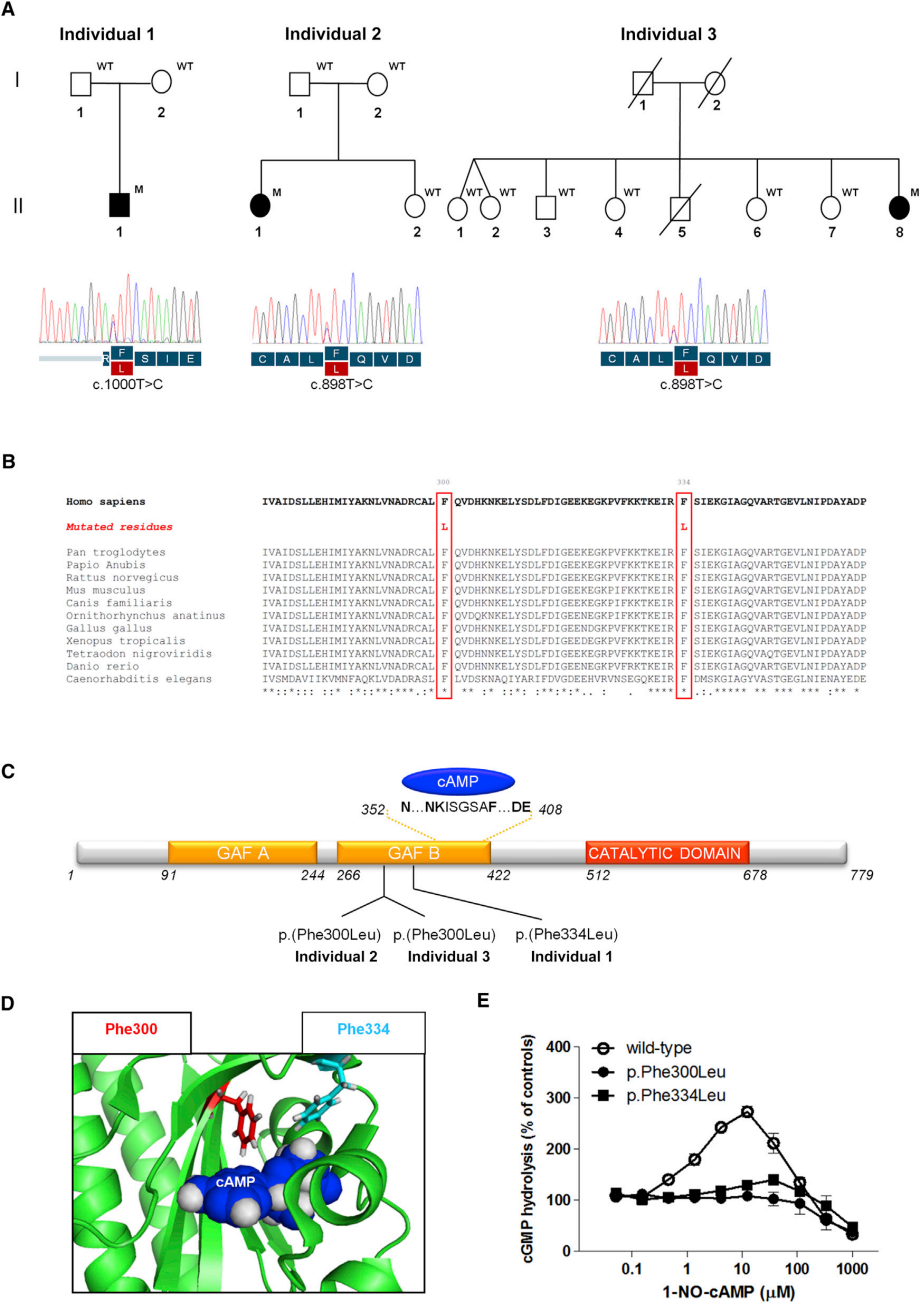
Pedigrees, *PDE10A* Mutation Analysis, Interspecies Alignment, Schematic Representation of PDE10A, In Silico Modeling of the 3D Structure of the PDE10A GAF-B Domain, and Functional Studies of the Identified PDE10A Substitutions (A) Pedigrees of the three individuals carrying the de novo *PDE10A* mutations and Sanger sequencing confirmation of the mutations. The following abbreviations are used: WT, homozygous wild-type alleles; and M, heterozygous *PDE10A* mutations. (B) Interspecies alignment performed with Clustal Omega shows the complete conservation down to invertebrates of the amino acid residues affected by the substitutions. Asterisks indicate invariant residues (full conservation), whereas colons and periods represent strong and moderate similarities, respectively. (C) A schematic representation of PDE10A shows its organization in three domains: the regulatory GAF-A and GAF-B domains in the N-terminal portion of the protein and the catalytic domain in the C terminus. The p.Phe300Leu and p.Phe334Leu substitutions are both located in the GAF-B domain, which binds to cAMP. (D) In silico modeling of the 3D structure of the GAF-B domain binding pocket and its interaction with the cAMP (shown in blue) was generated with PDB: 2ZMF. The variant residues Phe300 and Phe334 and their aromatic side chains, located in the β1 and β3 sheets, respectively, forming the floor of the cAMP binding pocket, are shown in red and cyan, respectively. Both residues are located in very close proximity to the cAMP molecule and are therefore likely to play an essential role in nucleotide binding. (E) The p.Phe300Leu and p.Phe334Leu substitutions cause a loss of stimulatory effect of the GAF-B domain on PDE10A catalytic activity. The effect of cyclic nucleotides binding to the GAF-B domain on PDE activity was evaluated via measurement of the enzyme activity after WT and mutant PDE10As were incubated in the presence of various concentrations of 1-NO-cAMP and 70 nM [^3^H]cGMP. Each data point represents the mean ± SEM of three independent experiments.

It is noteworthy that MRI of individual 1 (II-1 in Figure 2A; aged 11 years when scanned) showed slight swelling of the striata (Figure 1A) together with restricted diffusion (Figures 1B and 1C), suggesting an active disease process. Conversely, MRI of individual 2 (II-1 in Figure 2A; aged 22 years when scanned) demonstrated modest atrophy of the putamina (Figure 1D) and normal diffusion (Figures 1E and 1F), suggesting a more advanced stage of disease. MRI of individual 3 (II-8 in Figure 2A; aged 53 years when scanned) was markedly degraded by movement artifacts but also showed T2 hyperintensity within the posterolateral putamina (Figure S1A), although it was less dramatic than in the two younger individuals. Interestingly, individual 3, who is currently 60 years old, developed levodopa-responsive parkinsonism with freezing and falls in the fifth decade. Imaging of the density of striatal dopamine reuptake transporters (i.e., with DaTscan) was bilaterally abnormal, consistent with nigrostriatal degeneration (Figure S1B).

The homogeneous clinical and radiological appearance of these individuals was suggestive of a common genetic entity. Yet, extensive genetic and biochemical diagnostic work-up—focused on a wide spectrum of genetic diseases, including BHC, metabolic disorders, and mitochondrial diseases—was unrevealing.

Next, whole-exome sequencing (WES) was performed in all three individuals, as well as in the unaffected parents of individuals 1 and 2. The study was approved by the local ethics committees (Commissie Mensgebonden Onderzoek Arnhem-Nijmegen of Radboud University Medical Center for individual 1 under the realm of diagnostic exome sequencing and University College London Hospitals project 06/N076 for individuals 2 and 3). Written informed consent was obtained for all individuals, after which DNA was extracted from peripheral lymphocytes according to standard protocols. WES was performed as previously described.^7^, ^8^ In brief, exomes were enriched with either the Agilent SureSelectXT Human All Exon 50 Mb Kit (individual 1) or Illumina’s Nextera Rapid Capture (individuals 2 and 3) and sequenced on SOLiD 5500XL (individual 1) or a HiSeq 3000 (individuals 2 and 3) to an average sequence depth of 91×; on average, 89% of targets were covered at least 20×. Subsequently, variants were called and annotated with a custom in-house diagnostic pipeline^7^ (individual 1) or ANNOVAR^9^ (individuals 2 and 3). Given the sporadic occurrence of the phenotypes, filtering of variants focused on de novo dominant or recessive mutations (Figure 2A). Under the assumption that all three individuals would harbor a mutation in the same gene, we determined the overlap for putatively damaging mutations (defined as nonsense, frameshift, canonical splice-site, or predicted damaging missense mutations on the basis of CADD scores^10^ > 20) with a minor allele frequency < 1% in the Exome Aggregation Consortium (ExAC) Browser^11^ and an in-house database containing >10,000 individuals.

We identified only a single gene, *PDE10A* (MIM 610652; GenBank: NM_001130690.2), containing a variant in all three individuals. In individual 1, the heterozygous variant c.1000T>C was identified and predicted to result in p.Phe334Leu. Individuals 2 and 3 carried the same heterozygous variant, c.898T>C, which is predicted to result in p.Phe300Leu. Notably, the family-based sequencing approach of individuals 1 and 2 directly indicated that both *PDE10A* mutations had occurred de novo (Figure 2A). The parents of individual 3 are deceased, but the DNA of six unaffected siblings was available for testing, and none of them harbored the mutation. Further haplotype analysis using three microsatellites spanning the *PDE10A* locus identified the four parental haplotypes and revealed that the individual harboring the mutation shares one of the haplotypes with two siblings and shares the other with three other siblings, strongly suggesting a de novo occurrence of the mutation in this individual as well (Figure 2A and Figure S2). Analysis of the same three microsatellites in the family of individual 2, who carries the same de novo *PDE10A* change, indicated that the mutation arose on a different background haplotype (Figure S2). De novo mutations in *PDE10A* have not been observed in control individuals,^12^, ^13^, ^14^, ^15^, ^16^ and neither p.Phe300Leu nor p.Phe334Leu is listed in the ExAC Browser (last accessed in November 2015) or in-house databases, together containing ∼75,000 individuals. *PDE10A* has a residual variation intolerance score^17^ of −0.98, indicating that it belongs to the top 8.8% of the human genes most intolerant to genetic variation. Furthermore, constraint metrics reported in the ExAC Browser indicate that *PDE10A* is intolerant to both loss-of-function (probability of loss-of-function intolerance = 1.00) and missense (*Z* score = 3.78) mutations.^18^ Interspecies alignment of protein sequences generated with Clustal Omega^19^ revealed that the substitutions affect amino acid residues that are completely conserved down to invertebrate species (Figure 2B).

**Figure 3:**
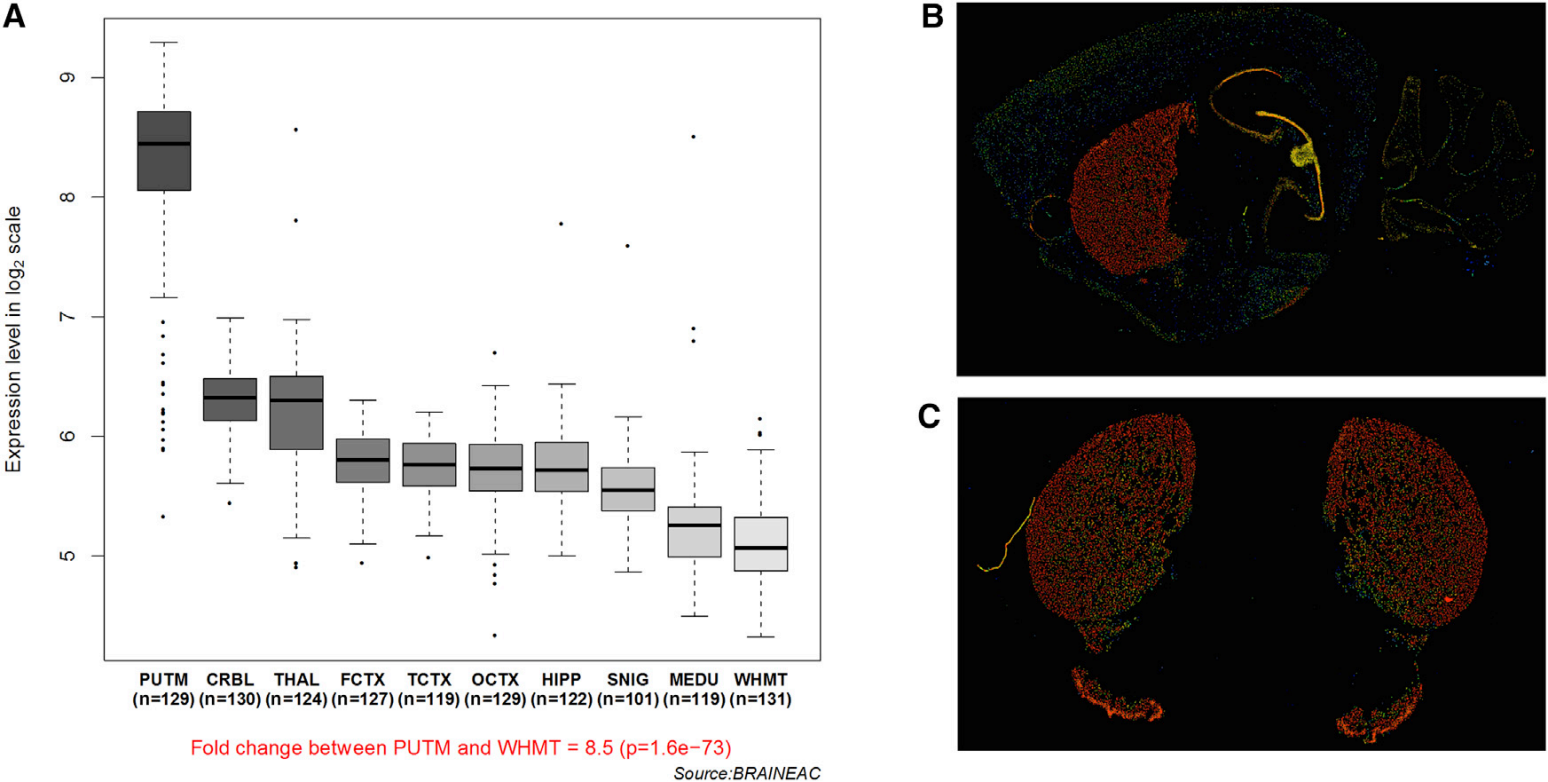
Summary of *PDE10A* mRNA Expression in the Human and Mouse Brain (A) Boxplots of *PDE10A* mRNA expression levels in ten adult brain regions (source: BRAINEAC, see Web Resources). The expression levels are based on exon array experiments and are plotted on a log_2_ scale (y axis). This dataset was generated with Affymetrix Exon 1.0 ST arrays and brain tissue originating from 134 control individuals, collected by the Medical Research Council Sudden Death Brain and Tissue Bank and the Sun Health Research Institute, an affiliate of Sun Health Corporation.^20^ This plot shows significant variation in *PDE10A* expression across the ten brain regions analyzed, such that expression is higher in the putamen than in any other region. Abbreviations are as follows: PUTM, putamen; FCTX, frontal cortex; TCTX, temporal cortex; OCTX, occipital cortex; HIPP, hippocampus; SNIG, substantia nigra; MEDU, medulla (specifically the inferior olivary nucleus); WHMT, intralobular white matter; THAL, thalamus; CRBL, cerebellar cortex; and N, number of samples analyzed for each brain region. (B and C) *PDE10A* expression in the mouse brain in (B) sagittal and (C) coronal sections. *PDE10A* was very highly and selectively expressed in the striata and in the olfactory tubercula. Images were obtained from the Allen Mouse Brain Atlas (©2015 Allen Institute for Brain Science). Expression intensity is color coded and ranges from low (blue) to moderate (green, yellow) to high (red) intensity.

Next, we explored the regional expression of these genes in the normal adult human brain. To this end, we used microarray data (Affymetrix Exon 1.0 ST) from human post-mortem brain tissue collected by the UK Human Brain Expression Consortium as previously described.^20^ This analysis showed exceptionally high expression in the putamen (Figure 3A), which is consistent with the data available in the Allen Mouse Brain Atlas^21^ (Figures 3B and 3C) and previous work demonstrating high and selective *PDE10A* expression in human striatum at both the RNA and protein levels.^22^, ^23^

*PDE10A* encodes a member of the cyclic nucleotide (cNMP) phosphodiesterase (PDE) family, consisting of 21 different proteins grouped into 11 sub-families according to their affinity for the type of cNMP (cyclic adenosine monophosphate [cAMP] and/or cyclic guanosine monophosphate [cGMP]), cellular regulation, and tissue distribution.^24^ cNMPs are ubiquitously localized intracellular second messengers, which modulate a broad range of cellular functions and pathways.^25^ The intracellular concentration of cNMPs is tightly regulated through a fine balance between their synthesis (controlled by the activity of adenylyl and guanylyl cyclases^26^, ^27^) and degradation (mediated by PDEs, which hydrolyze the cNMPs into their corresponding monophosphate nucleoside^28^). PDEs function as homodimers (the dimer interface extends over the entire length of the molecule), and all share a highly similar catalytic domain located in the C-terminal portion of the protein. Conversely, the N-terminal portion, which contains the regulatory domains, is variable and differs between different PDE families.^29^ PDE10A contains two N-terminal domains, GAF-A and GAF-B, of which the latter binds to cAMP (Figure 2C).^30^, ^31^ cAMP binding increases the enzyme activity of the PDE10A catalytic domain.^32^ Although details of the GAF-B-dependent modulation of PDE10A enzyme activity are currently unclear, a general mechanism for the regulation of all PDEs has been postulated. In the non-activated state, the dimerized catalytic domains are packed against each other at the dimer interface, occluding the catalytic pockets. The binding of cAMP to the GAF-B domain induces a rotating movement of the catalytic domains, enabling substrate access to the catalytic pockets and a consequent increase in cNMP hydrolysis.^33^

The crystal structure of the PDE10A-GAF-B domain and its interaction with cAMP has been elucidated and consists of a six-stranded anti-parallel β sheet (β3, β2, β1, β6, β5, and β4) sandwiched between a three-helix bundle (α1, α2, and α5) on one side and three short helices (α3, α4, and 3_10_) on the other side.^34^ The cAMP molecule is almost completely buried deep in a tight binding pocket, the floor of which is formed by the β sheets and the roof of which is formed by two α helices (α3 and α4). Importantly, the amino acids Phe300 and Phe334 are located in the β1 and β3 sheets, respectively, and are positioned deep in the cAMP binding pocket of GAF-B in very close proximity to the cAMP molecule (Figure 2D). It is therefore postulated that the substitutions severely affect the morphology of the GAF-B binding pocket and/or alter its affinity for cAMP.

To assess the functional effect of the identified PDE10A substitutions in vitro, we investigated whether they affect (1) PDE basal enzyme activity and/or (2) the stimulatory effect on PDE catalytic activity mediated by cAMP binding to the GAF-B domain. cDNA for human PDE10A (GenBank: NM_001130690.2) was used as a template, and mutant constructs (c.898T>C [p.Phe300Leu] and c.1000T>C [p.Phe334Leu]) were inserted by site-directed mutagenesis. Wild-type (WT) and mutant constructs were cloned into the pcDNA3.1(+)neo vector (Thermo Fisher Scientific) and transfected into COS-7 cells (European Collection of Authenticated Cell Cultures). In vitro PDE enzyme activity was measured with a scintillation-proximity-assay (SPA)-based method.^35^ In this assay, the product of the PDE reaction, either [^3^H]-labeled AMP or GMP, binds directly to yttrium silicate PDE SPA beads (GE Healthcare), resulting in light emission. Reactions for kinetic studies were conducted with a mixture of [^3^H]-labeled and unlabeled cAMP or cGMP together with either WT or mutant PDE10A-expressing COS-7 cell membrane fractions. These experiments showed no significant difference between WT and mutant PDE10As (Figure S3), suggesting that both p.Phe300Leu and p.Phe334Leu do not substantially affect basal PDE10A enzyme activity.

We then explored whether the identified substitutions affect the stimulatory properties of cAMP binding to the GAF-B domain. We conducted experiments by using only [^3^H]cGMP as a substrate (to avoid the binding of [^3^H]cAMP substrate to the GAF-B domain) and the cAMP analog 1-NO-cAMP (Biolog Life Science Institute), whose selectivity for the GAF-B domain over the catalytic site is ∼28× higher than that of cAMP (247-fold for 1-NO-cAMP versus 8.7-fold for cAMP).^35^ These experimental conditions were chosen because, on the one hand, cAMP activates PDE10A enzyme activity via its binding to GAF-B, and on the other hand, cAMP competes at the catalytic domain with radio-labeled substrates and thus inhibits their degradation.^35^ 1-NO-cAMP markedly increased (approximately 2.7-fold over the basal levels) the enzyme activity of WT PDE10A, whereas this effect was almost completely lost for both mutant PDE10As (Figure 2E). These experiments demonstrate that p.Phe300Leu and p.Phe334Leu severely affect the positive regulatory mechanism of cAMP binding to the GAF-B domain on PDE catalytic activity.

PDEs have previously been implicated in the pathogenesis of neurodegenerative disorders, such as Parkinson disease and Huntington disease.^36^ Mutations in *PDE8B* (MIM: 603390), a gene highly expressed in the brain and especially in the putamen, causes autosomal-dominant striatal degeneration (ADSD [MIM: 609161]), a disease that clinically manifests with adult-onset parkinsonism.^37^, ^38^ Although the reported MRI abnormalities observed in subjects with ADSD are slightly different from those observed in our individuals, it is striking that both diseases are caused by alterations in PDEs, which lead to clearly visible, largely symmetric, striatal MRI signal abnormalities. Furthermore, the fact that two PDEs are now directly linked to a basal ganglia disease might point toward a crucial role of PDEs in these types of disorders. The latter is of great interest given the pharmacological potential to manipulate PDE activity. Given its high and selective presence in striatal MSNs, PDE10A is a primary target in pharmacological research for diseases where dysregulation of striatal circuits is believed to be crucial (e.g., psychosis, Huntington disease, substance abuse, and Parkinson disease).^39^

According to the classic model of basal ganglia motor circuits, chorea mainly results from dysregulation of MSN activity.^40^ Importantly, modulation of MSN activity is largely dependent on cAMP signaling.^41^ cAMP synthesis, and thus indirectly its signaling, is promoted by stimulation of the G-protein-coupled dopamine receptor D1 and adenosine receptor A2, whereas synthesis is inhibited by dopamine stimulation of dopamine receptor D2.^42^ The G protein Gα_olf_ positively couples receptors D1 and A2 to the activation of adenylate cyclase 5 (AC5), the main molecule responsible for cAMP production in MSNs.^43^ Interestingly, mutations in the genes encoding Gα_olf_ (*GNAL* [MIM: 139312]) and AC5 (*ADCY5*) have been identified as a cause of primary dystonia^44^ and chorea,^45^, ^46^ respectively.

Mechanistically, *ADCY5* mutations seem to increase AC5 activity and consequently cause raised intracellular cAMP levels in cellular models.^47^ Given that pathogenic mutations in both *PDE10A* and *ADCY5* cause chorea (even though PDE10A and AC5 exert opposite effects on cAMP levels), one would expect that the p.Phe300Leu and p.Phe334Leu variants exert a deleterious effect on PDE enzyme activity. Recent studies have suggested that PDE10A has two functional states: “active” and “super-active.”^32^, ^48^ In the presence of high intracellular levels of cAMP, its binding to the GAF-B domain would stimulate the PDE catalytic activity, switching PDE10A from the “active” to the “super-active” state. In light of this, PDE10A might function as a “brake” for MSN activation. Our functional studies showed that pathogenic *PDE10A* mutations located in the GAF-B domain severely disrupt this positive regulatory mechanism without affecting the basal PDE enzyme activity. These mutations might therefore have a strong impact on the in vivo regulation of MSN activity, especially when MSNs are activated by high levels of cAMP. Given the homodimerized structure of PDE10A, the mutant proteins could exert a dominant-negative effect on the activity of the WT protein.

In conclusion, we have demonstrated that de novo dominant mutations in *PDE10A* are the cause of a unique movement disorder characterized by benign childhood-onset chorea and typical MRI abnormalities of the striatum. Of note, screening of a cohort of ∼60 individuals with a BHC-like syndrome and lacking mutations in *NKX2-1* (clinically resembling subjects with *PDE10A* mutations but with normal brain MRI ) did not reveal any additional mutations in *PDE10A*. The latter suggests that *PDE10A*-related chorea might represent a distinct genetic clinico-radiological entity. Mutational screening of additional cohorts with such MRI abnormalities is warranted for further defining the clinical spectrum associated with *PDE10A* mutations. Furthermore, it will be important to establish whether the observation of parkinsonism with nigrostriatal degeneration in individual 3 is coincidental or whether individuals with de novo *PDE10A* mutations are also at an increased risk of developing degeneration of nigral neurons. In this regard, recent work has demonstrated that striatal reduction of PDE10A levels is associated with the duration and severity of Parkinson disease.^49^ With the previous discoveries of mutations in *GNAL*, *PDE8B*, and *ADCY5*, and now *PDE10A*, there is accumulating evidence that intracellular cAMP signaling in striatal MSNs is crucial for normal activity of basal ganglia circuitry and that disruptions thereof play an important role in the pathophysiology of movement disorders. Our results highlight pharmacological manipulation of cAMP levels in MSNs as a promising therapeutic strategy for the treatment of chorea and other movement disorders.

## Conflicts of Interest

The work was partly funded by Takeda Pharmaceutical Company Limited, which provided support in the form of salaries for some of the authors (K.N., K.S., C.H.D., and H.K.) but did not have any additional role in the study design, data collection and analysis, decision to publish, or preparation of the manuscript.

## Figures and Tables

**Table 1:** Genetic, Clinical, and Radiological Findings of Individuals with *PDE10A* Mutations

	**Individual 1**	**Individual 2**	**Individual 3**
Age at most recent clinical examination	11 years	22 years	60 years
Gender	male	female	female
Descent	European (Dutch)	European (British)	European (British)
CADD score^a^	31.0	28.7	28.7

***PDE10A* Mutation**			

Genomic position (GRCh37)	chr6: 165,829,768 A>G	chr6: 165,832,223 A>G	chr6: 165,832,223 A>G
cDNA (GenBank: NM_001130690.2)	c.1000T>C	c.898T>C	c.898T>C
Protein	p.Phe334Leu	p.Phe300Leu	p.Phe300Leu
Inheritance	de novo	de novo	de novo^b^

**Neurology**			

Developmental milestones	normal	normal	normal
Cognition	normal	normal	normal
Chorea (age of onset)	+ (5)	+ (8)	+ (5)
Other	no	anxiety	adult-onset parkinsonism

**MRI**			

Bilateral striatal hyperintensities	+	+	+
Bilateral striatal swelling	+	−	−
Restriction of diffusion	+	−	NA
Bilateral striatal atrophy	−	+	+

Abbreviations are as follows: +, present; −, absent; and NA, not available.

a A score ≥ 20 indicates that the variant is predicted to be the among the 1% most deleterious substitutions in the protein-coding parts of the human genome.

b Haplotype analysis in unaffected siblings suggests the de novo occurrence of the mutation in individual 3.

## Web Resources

The URLs for data presented herein are as follows:

Allen Mouse Brain Atlas, http://mouse.brain-map.org/
BRAINEAC, http://www.braineac.org/
CADD, http://cadd.gs.washington.edu/home
Clustal Omega, http://www.ebi.ac.uk/Tools/msa/clustalo/
Exome Aggregation Consortium Browser, http://exac.broadinstitute.org/
Genic Intolerance, http://genic-intolerance.org/
OMIM, http://www.omim.org/
RefSeq, http://www.ncbi.nlm.nih.gov/refseq/
UK Human Brain Expression Consortium, http://www.braineac.org/

## Supplemental Data

Document S1. Figures S1–S3

Document S2. Article plus Supplemental Data
