# Supplementary material for: De Novo Mutations in PDE10A Cause Childhood-Onset Chorea with Bilateral Striatal Lesions
Source: Am J Hum Genet. 2016 Apr 7;98(4):763–71. doi: 10.1016/j.ajhg.2016.02.015 (PMC4833291; doi:10.1016/j.ajhg.2016.02.015)
Supplement: Document S1. Figures S1–S3 [file mmc1.pdf]

The American Journal of Human Genetics, Volume *98*

Supplemental Data

De Novo Mutations in *PDE10A* Cause Childhood-Onset Chorea with Bilateral Striatal Lesions

Niccolò E. Mencacci, Erik-Jan Kamsteeg, Kosuke Nakashima, Lea R'Bibo, David S. Lynch, Bettina Balint, Michèl A.A.P. Willemsen, Matthew E. Adams, Sarah Wiethoff, Kazunori Suzuki, Ceri H. Davies, Joanne Ng, Esther Meyer, Liana Veneziano, Paola Giunti, Deborah Hughes, F. Lucy Raymond, Miryam Carecchio, Giovanna Zorzi, Nardo Nardocci, Chiara Barzaghi, Barbara Garavaglia, Vincenzo Salpietro, John Hardy, Alan M. Pittman, Henry Houlden, Manju A. Kurian, Haruhide Kimura, Lisenka E.L.M. Vissers, Nicholas W. Wood, and Kailash P. Bhatia

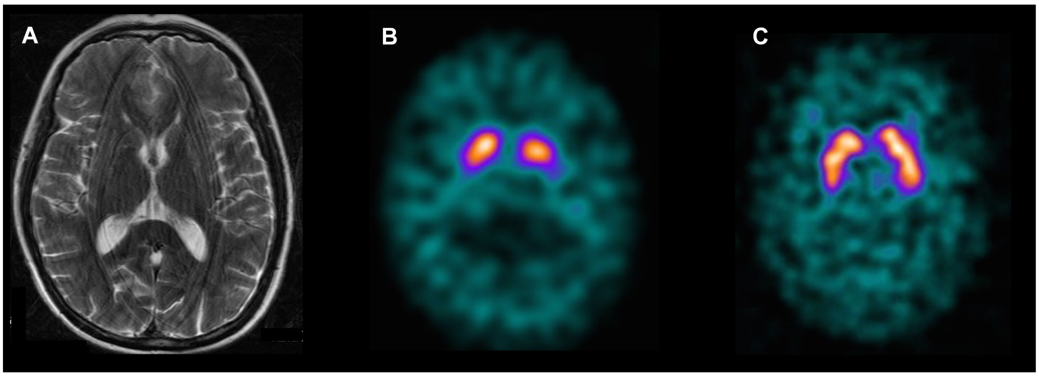

Figure S1. Brain MRI and single photon emission computed tomography (SPECT) dopamine reuptake transporter (DAT)-scan images in case 3. (A) Albeit markedly degraded by movement artefacts, axial MR images showed bilateral T2 hyperintensity within the posterolateral putamina. (B) Dopaminergic striatal innervation was evaluated as DAT density by means of ^{123}I-FP-CIT SPECT. The scan shows marked bilateral reduction of tracer uptake in the striatum, consistent with bilateral nigrostriatal dopaminergic denervation. (C) Normal DAT-scan from an age-and sex matched subject.

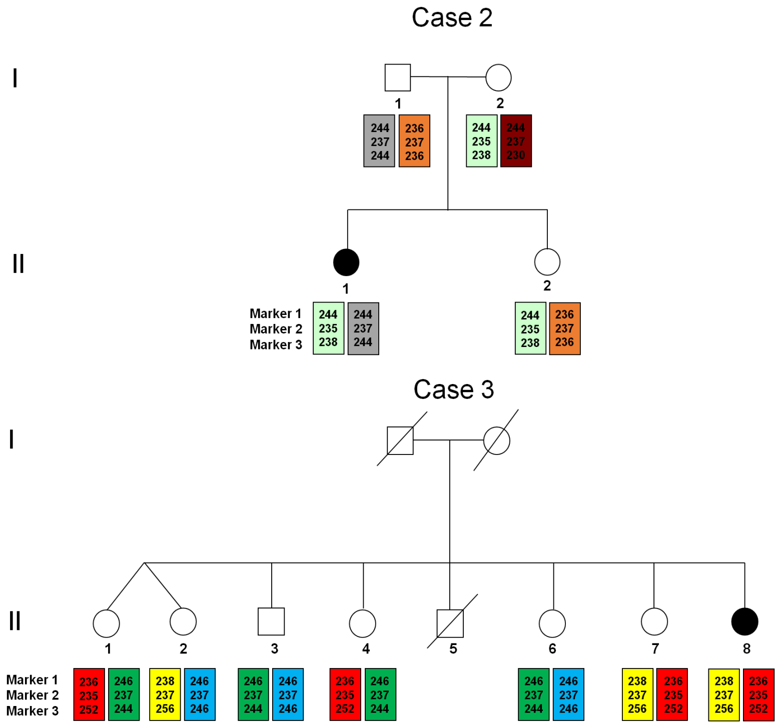

Figure S2. Haplotype analysis in the families of cases 2 and 3. Haplotype analysis was performed to test whether the c.898C>T mutation had arisen on the same genetic background in cases 2 and 3 and to unveil the *de novo* occurrence of the c.898C>T mutation identified in case 3. Three microsatellites (di-nucleotide repeats) surrounding the *PDE10A* locus (Marker 1 – chr6:166069747-166069785; Marker 2 – chr6:165862198-165862227; Marker 3 -

chr6:165839259-165839288; primers available upon request) were sized up in all available relatives of the two cases. The four parental haplotypes were reconstructed in both families (each haplotype defined by a different color). Cases 2 and 3 did not share the haplotype encompassing the c.898C>T variant, suggesting the mutations arose on different haplotype backgrounds. Furthermore, haplotype analysis indicates that case 3, who carries the *PDE10A* c.898T>C variant, shares one of the allele (marked in yellow) with two unaffected siblings (II-2, II-7) whereas the other allele (marked in red) is shared with three unaffected siblings (II-1, II-4 and II-7). Of note, Sanger sequencing showed that all unaffected siblings are homozygous for the wild-type allele. These data strongly support the *de novo* occurrence of the mutation in case 3.

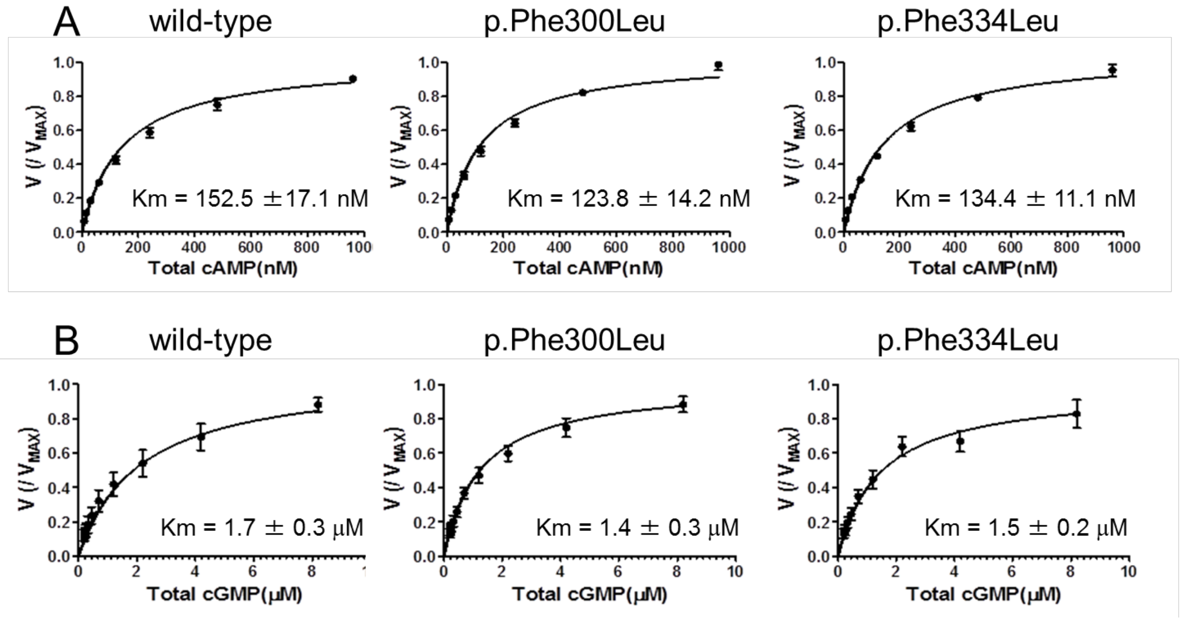

Figure S3. Enzyme Kinetics of Wild-Type and Mutant PDE10As. PDE10A enzymes was incubated at presence of mixture of unlabeled cAMP and [^{3}H]cAMP (A) or unlabeled cGMP and [^{3}H]cGMP (B) with the total concentration as indicated. To obtain the Michaelis–Menten constants (Km), the initial rates of the reaction were fitted to the following equations using GraphPad Prism (GraphPad Software, Inc., La Jolla, CA, US): V = Vmax [S] / (Km + [S]); where V is the initial velocity of the enzyme-catalyzed reaction, [S] is the substrate concentration, Vmax is the limiting reaction velocity at saturating substrate concentrations, and Km is the Michaelis–Menten constant (concentration of substrate at 1/2 of Vmax). Each data point represents the mean ± S.E.M. of five (for cAMP) and four (for cGMP) independent experiments. There was no statistically significant difference in Km values among the wild-type and the mutant PDE10As ($p > 0.05$ by Dunnett's test compared with wild-type).
